# A Novel Promising Strain of *Trichoderma evansii* (WF-3) for Extracellular *α*-Galactosidase Production by Utilizing Different Carbon Sources under Optimized Culture Conditions

**DOI:** 10.1155/2014/461624

**Published:** 2014-07-13

**Authors:** Aishwarya Chauhan, Nikhat Jamal Siddiqi, Bechan Sharma

**Affiliations:** ^1^Department of Biochemistry, Faculty of Science, University of Allahabad, Allahabad 211002, India; ^2^Department of Biochemistry, King Saud University, Riyadh, Saudi Arabia

## Abstract

A potential fungal strain of *Trichoderma* sp. (WF-3) was isolated and selected for the production of *α*-galactosidase. Optimum conditions for mycelial growth and enzyme induction were determined. Basal media selected for the growth of fungal isolate containing different carbon sources like guar gum (GG), soya bean meal (SM), and wheat straw (WS) and combinations of these carbon substrates with basic sugars like galactose and sucrose were used to monitor their effects on *α*-galactosidase production. The results of this study indicated that galactose and sucrose enhanced the enzyme activity in guar gum (GG) and wheat straw (WS). Maximum *α*-galactosidase production (213.63 UmL^−1^) was obtained when the basic medium containing GG is supplemented with galactose (5 mg/mL). However, the presence of galactose and sucrose alone in the growth media shows no effect. Soya meal alone was able to support *T. evansii* to produce maximum enzyme activity (170.36 UmL^−1^). The incubation time, temperature, and pH for the maximum enzyme synthesis were found to be 120 h (5 days), 28°C, and 4.5–5.5, respectively. All the carbon sources tested exhibited maximum enzyme production at 10 mg/mL concentration. Among the metal ions tested, Hg was found to be the strongest inhibitor of the enzyme. Among the chelators, EDTA acted as stronger inhibitor than succinic acid.

## 1. Introduction

Nature is enriched with different carbon sources, which were utilized as food by different organisms. Among different organisms, the fungal species were the most potent source for production of different extracellular enzymatic machinery for utilizing these carbon sources as a food product. They cause breakdown of the complex polymers of carbohydrates into its oligomeric and monomeric units. The high yielding and stable strains may be isolated by using a media containing inducers of the products and devoid of repressors. In general, no defined medium was established for the best production of any metabolite because the genetic diversity present in different microbial sources causes each organism or strain to have its own special conditions for maximum product formation. Therefore, it is essential to have a detailed investigation on growth and metabolite production pattern of newly isolated microbial strain under different environmental conditions to achieve maximum production benefits. Currently different fungal enzymatic machineries are being utilized by many food, feed, pharma, and biotechnological industries for the synthesis of desired products. Among these, glycoside hydrolases (glycosidases or carbohydrases; EC 3.2.1) are enzymes that catalyze hydrolytic cleavage of O-glycoside bond and are classed among enzymes of carbohydrate catabolism. For many decades, glycoside hydrolases have been subjects of many biochemical studies [1, 2].

All known glycoside hydrolases are divided into 90 families. Among these members, the action of *α*-galactosidase (*α*-Gal) is uniquely designed to liberate free galactose from the nonreducing end of the substrates. Hence, the systematic name of this glycosidase is *α*-galactoside galactohydrolase (EC 3.2.1.22). According to their substrate specificities, *α*-galactosidases can be divided into two groups [1]. The first group contains *α*-galactosidase active only on oligosaccharides with low degree of polymerization, for example, melibiose, raffinose, stachyose, and short fragments of galactoglucomannans. These enzymes are usually very active on artificial substrates like aryl pNp-*α*-D-galactopyranosides. The second group consists of enzymes active on polymeric substrates. *α*-Gal activity has been reported from animals, plants, and microbes [2, 3], but humans and monogastric animals lack *α*-Gal in their digestive tract [4].

The *α*-galactosidases are reported to be extensively synthesized in the microorganisms including bacteria and fungi. As might be expected, research in this area has centered on sources of *α*-Gal that offer the greatest economic potential. Filamentous fungi have been extensively employed on an industrial scale for many decades for the production of number of enzymes. The fungal galactosidases are the most suitable for technological applications mainly due to their extracellular localization, acidic pH optima, and broad stability profiles [5]. For example, *α*-galactosidases from* Trichoderma reesei* and* Aspergillus niger* have an optimal pH of 4.0-5.0 and optimal temperature of 50–60°C [6–8]. Several species of filamentous fungi produce extracellular *α*-galactosidases. They have been identified and purified from many* Aspergillus* sp.,* Trichoderma* sp.,* Penicillium* sp., and so forth [9–16]. *α*-Gal may have great potential in various applications [17–19]. Industrially, the *α*-galactoside removal is achieved by treatment of soy milk with a crude extract of *α*-Gal obtained from the fungus,* Aspergillus oryzae* [20, 21]. Another related application of *α*-Gal is in the form of a dietary supplement to overcome flatulence, bloating, and abdominal discomforts, widely known in pharmaceutical industries as Novozymes and Beano [21–23] in which the source of *α*-Gal is the fungus* Aspergillus niger*. In beet industry, this activity is used to remove raffinose from molasses in order to facilitate and improve the crystallization and yield of sucrose [24]. Galactooligosaccharides produced by transfer reaction of *α*-Gal can be used as a prebiotic in functional food [25]. Removal of a quantitative proportion of galactose moieties from GG by *α*-Gal improves the gelling properties of the polysaccharide and makes them comparable to those of locust bean gum [26].

The *α*-Gal is also of medical importance. The deficiency in the activity of lysosomal *α*-Gal A in human causes Fabry disease. Fabry disease is an X-linked inborn error of glycosphingolipid metabolism resulting from mutations in the *α*-Gal A gene. This gene encodes two enzymes: agalsidase-*α* (Replagal) and agalsidase-*β* (Fabrazyme) [21, 23]. Moreover, the enzymatic conversion of group B red blood cells to group O red blood cells* in vitro* is attained by the use of *α*-Gal from coffee beans [27]. Such varied applications of *α*-Gal encouraged us to explore isolation of more potential strains of fungal species from soil producing extracellular *α*-galactosidases, in order to exploit their applications in different food, feed, and medical industries. In progression of our investigation, we isolated and examined a new producer of *α*-Gal, that is,* Trichoderma* sp., to introduce more potent and less pathogenic strains for their industrial utilization.

The monovalent and divalent metal ions have been reported to modulate the function of *α*-galactosidases activity from different fungal species. Their effects have been shown in species specific manner. In addition, the effects of chelators, that is, EDTA and succinic acid have also been reported to evaluate their role in regulation of enzyme activity. However, the impact of these metal ions and chelators on the activity of *α*-galactosidases produced by* Trichoderma* sp. has not been worked out systematically.

The present study is an endeavour to optimize secondary culture media conditions for *α*-galactosidase production, secreted by* Trichoderma evansii* (WF-3). As reported earlier [28], this fungal species was isolated from soil which appeared to be a novel source for *α*-Gal production. Trichoderma is a member of the Ascomycota, the largest group of fungi. Asexual reproduction occurs through the production and germination of asexual conidia [20]. Trichoderma has been exploited in many industries including paper, textile, biofuel, and agriculture due to its prolific secretion of degrading enzymes and biocontrol activities [29–32]. In this paper, we have presented culture characterization and primary identification of a fungal species which has immense potential of secretion of *α*-Gal. Various culture conditions such as incubation time, pH, temperature, and substrate concentration have been standardized to obtain maximum production of the enzyme in culture filtrate. The results from this study could be utilized in pulp and paper industries, treatment of legume based foods, and clinical medicines.

## 2. Materials and Methods

### 2.1. Chemicals

p-nitrophenyl-*α*-D-galactopyranosides (pNPGal), synthetic substrate for screening of*α*-galactosidase activity, and para-nitrophenol (pNP), chromogenic substrate for standard preparation, were purchased from Sigma Chemical Co. (St. Louis, MO, USA); Folin-Ciocalteu's phenol reagent and sodium carbonate were from Merck Chemical Supplies (Darmstadt, Germany). All other chemicals used were of analytical grade.

### 2.2. Isolation of Fungi

**Figure 1:**
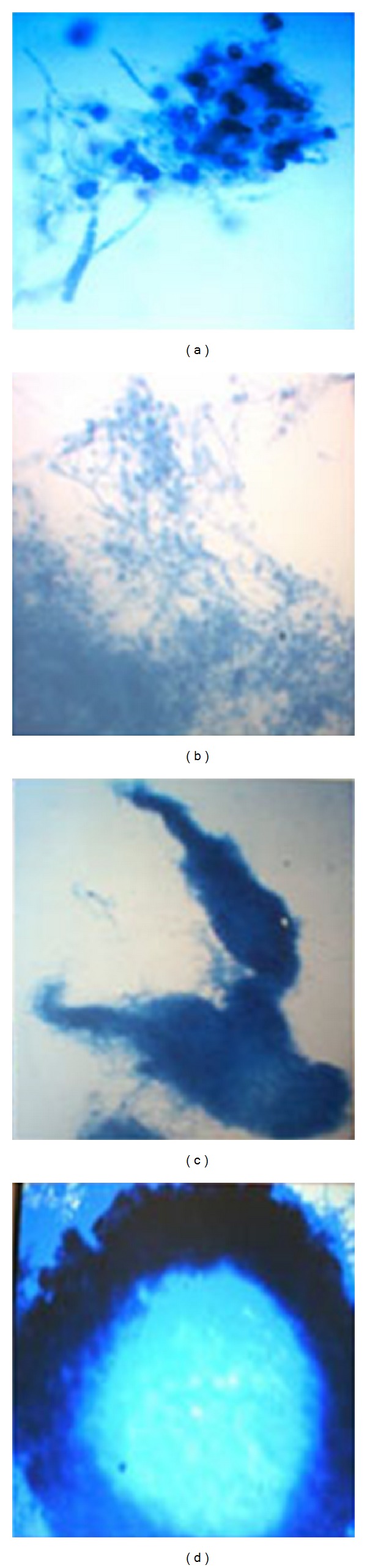
Growth pattern of* T. evansii* in the presence of different carbon sources in the culture medium containing potato-dextrose-agar (PDA, solid medium) (a). After 7 days of growth of the fungal culture in broth containing different carbon sources such as soya meal (SM) (b), guar gum (GG) (c), and wheat straw (WS) (d). The corresponding fungal floccules were stained with cotton blue and viewed under compound microscope for their comparison with that grown in PDA.

Selected fungi, that is,* Trichoderma evansii* (WF-3), were isolated from rhizospheric soil of* Phyllanthus emblica*(aamla) collected from the local garden of Sagar, India [33]. They were isolated by direct soil plate method [34] and identified accordingly [35–37]. Pure cultures were maintained on PDA slants. Growth pattern of* T. evansii* in the presence of different carbon sources in the culture medium containing potato-dextrose-agar (PDA solid medium) and other carbon sources has been shown in Figure 1(a). After 7 days of growth of the fungal culture in broth containing different carbon sources such as soya meal (SM) Figure 1(b), guar gum (GG) Figure 1(c), and wheat straw (WS) Figure 1(d); the concerned enzyme activities are demonstrated. The corresponding fungal floccules were stained with cotton blue and viewed under compound microscope for their comparison with that grown in PDA (Figure 1).

### 2.3. Culture Medium and Conditions

A basal solution consisted of KH_2_PO_4_ (7.0 g L^−1^), K_2_HPO_4_ (2.0 g L^−1^), MgSO_4_ *·*7H_2_O (0.1 g L^−1^), (NH_4_)_2_SO_4_ (1.0 g L^−1^), yeast extract (0.6 g L^−1^) in tap water to a volume of 1000 mL, and 1% (w/v) dry contents of each of the selected substrates such as extracts of guar gum (GG), soya bean meal (SM), and wheat straw (WS). The galactose and sucrose were added separately 1% (w/v) and in combination with selected carbon sources (each with 0.5% w/v) into the basal medium. For each particular treatment, 250 mL Erlenmeyer conical flasks were prepared containing 50 mL of basal or optimized media, sterilized at 121°C for 20 min under 1.5 atmospheric pressure, and cooled to room temperature. On cooling, they were inoculated by addition of three pellets of heavily sporulating fungi from 4- to 5-day-old cultures, were picked up through cork borer (1* ×* 1 cm in diameter), and were added to Erlenmeyer flasks each of 250 mL capacity (10^5^ spores mL^−1^, if otherwise not stated). The cultivation was carried out on rotary shaker (120 rpm) and incubated at 28°C for 11 days. The *α*-Gal activity and protein content of the culture filtrate were determined in the culture filtrates from each set of experiments. After 11 days of growth of the fungal culture in broth containing different carbon sources, the corresponding fungal floccules were stained with cotton blue and viewed under compound microscope for their comparison with that grown in PDA.

### 2.4. Enzyme Activity Assay

The *α*-Gal activity assay was carried out in test tubes by the modified version of the method by using p-nitrophenyl-*α*-D-galactopyranosides (pNPGal) as substrate. The assay system contained 0.2 mL of 0.05 M citrate buffer (pH 5.0), 0.3 mL of 1.0 mM pNPGal solution, and 100 *μ*L of enzyme preparation. The reaction was started by addition of pNPGal. The reaction mixture was incubated for 10 min at 50°C and was stopped by the addition of 0.4 mL of 1.0 M sodium carbonate solution. The amount of p-nitrophenol (pNP) released was determined spectrophotometrically using UV-Visible double beam spectrophotometer (Spectrascan UV 2700) at 405 nm. One unit (U) of enzyme was defined as the amount of *α*-Gal enzyme which liberates 1 *μ*mol of pNP per minute under the given assay conditions.

### 2.5. Protein Estimation

The extracellular protein content excreted in the culture filtrate by each of the fungal strains was determined by the method as described [38] using bovine serum albumin (BSA) as a standard. The culture filtrate without any fungal inoculums was used as a control.

### 2.6. Optimization of Incubation Time for Maximum Enzyme Production

Culture media containing different carbon sources have been incubated up to 11 days in shaking flask conditions at 28°C. At the interval of every 24 h, the aliquots of culture filtrate from each media containing GG, SM, and WS with and without galactose and sucrose were collected in 2 mL of microcentrifuge tubes with the help of micropipette of 1 mL capacity. This culture filtrate was then centrifuged at 3000 rpm for 3 min and the supernatant was used for estimation of *α*-Gal activity and the protein secreted extracellularly in each culture media.

### 2.7. Effect of Different Carbon Substrates Alone and in Combination with Galactose (GAL) and Sucrose (SUC) on Fungal Enzyme Synthesis

The powder forms of SM, GG, and WS were used in the screening experiment to choose the most suitable substrate for maximum *α*-Gal production.

Carbon sources used for enzyme production were added with galactose and sucrose separately in equal quantity, that is, 5 mg/mL each, in order to check the effect of addition of these two sugars on enzyme secretion in media. When galactose and sucrose were used individually, 10 mg/mL concentration of each was used in the medium.

### 2.8. Effect of pH and Temperature on Enzyme Synthesis and Secretion

The effect of pH on the synthesis and secretion of fungal enzyme was determined by adjusting the culture media at various pH (3.5, 4.5, 5.5, 6.5, and 7.4) with the help of 0.1 N NaCl and 0.1 N HCl. The culture media were then incubated at optimized temperature for optimized period. The optimum pH for maximum enzyme activity was determined using standard assay conditions.

In order to determine the optimum temperature required for maximum production of the fungal enzyme, the culture media containing different carbon sources and the fungi were incubated at two different temperatures (28 and 40°C) up to 6 days. The residual enzyme activity was determined after days 5 and 6 using standard assay conditions.

### 2.9. Effect of Substrate Concentration on Enzyme Synthesis and Secretion

Three substrate concentrations, that is, 6, 10, and 14 mg/mL, were used into the culture media (50 mL) in order to determine the optimum utilization of the substrate by the fungal species to produce maximum enzyme activity in the medium.

### 2.10. Effect of Different Inhibitors and Chelators on Enzyme Activity* In Vitro*

The effects of different metal ions like HgCl_2_, CuCl_2_, MgCl_2_, and AgNO_3_ and chelators such as EDTA and succinic acid on *α*-Gal activity were determined* in vitro* by treating the enzyme with their varying concentrations. Other optimal experimental conditions were kept constant.

## 3. Results and Discussion

The isolated and identified* Trichoderma* strain, that is,* Trichoderma evansii* (WF-3), displayed ability of secretion of an extracellular *α*-Gal. Another species of this fungus,* Trichoderma reesei*, has already been reported to contain variable amounts of this enzyme bound to the cell wall [35]. Therefore, all further investigations on the formation of *α*-Gal were done by measuring the total amount of secreted enzyme.

The following sections deal with the results obtained on the standardization of culture media conditions such as incubation time, pH, temperature, different carbon sources, and their concentrations along with effect of inducers like galactose and sucrose for detection of maximum enzyme excretion by respective fungal strain in growth media.

### 3.1. Effect of Incubation Time and Different Carbon Sources on the *α*-Gal Synthesis

**Figure 2:**
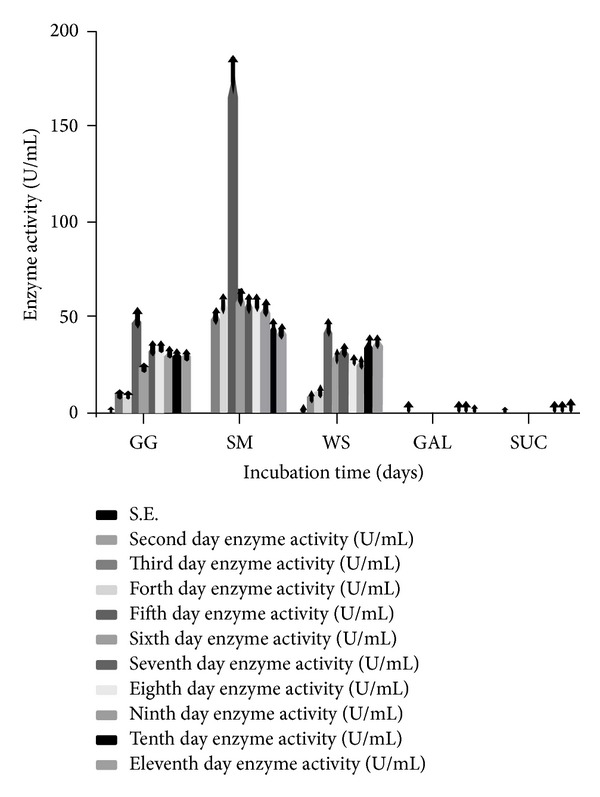
Effect of different carbon sources on the level of *α*-Gal activity (U/mL) secreted by* T. evansii* (WF-3) up to 11 days. GG = guar gum, SM = soya bean meal, WS = wheat straw, GAL = galactose, and SUC = sucrose. The experiments have been conducted three times and the values are shown as mean ± SD. Blank bar = not detected (for first day enzyme activity).

In order to find out the optimum incubation time for maximum enzyme production, *α*-Gal activity was determined in the culture filtrate after interval of every 24 h. The media containing different substrates like GG, SM, and WS and also in combination with galactose and sucrose were incubated at 28°C in shaking flask (120 rpm). The effect of different incubation periods on *α*-Gal production using basal fermentation medium is shown in Figure 2. The optimum production was obtained at the fifth and sixth day of incubation period; maximum activity was shown by WF-3 fungal strain into the culture filtrate (213.63 U/mL) in media containing GG + GAL followed by media containing SM (170.36 U/mL) and others. However, the media containing GG + SUC and WS + SUC registered minimum enzyme activity on days 5 and 6. The longer incubation of up to 11 days showed decreasing trend in the enzyme activity (Figure 2). It has already been established that the microbial production of *α*-Gal varies with the growth rate [35] as the activity increases with increase in biomass concentration [39]. Similar observations have been reported by Anisha and Prema [40], where they have shown that the biomass increased with the increasing period of incubation with the increase in enzyme production. The highest enzyme production for AGP47 and AGP42 was reported after fourth and sixth day of incubation, respectively, after which cell mass declined and also the enzyme production [40]. Similar to our results, El-Gindy et al. [41] reported that the sixth day of incubation was the best for the experimental fungi where* A. awamori* produced maximum *α*-Gal activity (2.172 U/g), while* A. carbonarius* reached maximum *α*-Gal production at incubation period of 6 days (2.280 U/g). The activity of *α*-Gal secreted by* A. awamori* and* A. carbonarius* showed reducing trend, the values being 1.6 U/mL and 1.8 U/mL, respectively, upon increasing duration of incubation after 7 days. The decline of total enzyme activity could be considered to be the result of inhibition of cellular functions and due to depletion of nutritional factors from the growth medium or deactivation of enzyme due to pH change or due to inducer exclusion or due to action of secreted fungal proteases.

### 3.2. Effect of Different Carbon Substrates Alone and in Combination with Galactose (GAL) and Sucrose (SUC) on Fungal Enzyme Synthesis

In preliminary experiments, the ability of* T. evansii* to produce *α*-Gal in the presence of three different cheap carbon sources such as GG, SM, and WS was investigated. Since *α*-Gal is present on the conidia of this fungus [42], we considered it to be possible that the enzyme may release galactose from GG, SM, and WS, thereby producing an inducer of further *α*-Gal biosynthesis. This hypothesis was previously applied by Kubicek et al. [43] as well by using locust bean gum, melibiose, raffinose, and other *α*-galactoside linked oligosaccharides. When carbon sources were used individually, the maximum enzyme production was obtained in the presence of SM followed by GG and WS with very less activity in presence of GAL and SUC as shown in Figure 2. The *α*-Gal secreted by* T. evansii* displayed maximum activity on the fifth day of incubation in the presence of each of the SM (170.36 U/mL), GG (48.66 U/mL), and WS (43.25 U/mL) as carbon source (Figure 2). Similar results were obtained by other workers [43] as well when culture filtrates of* A. fumigatus* were supplemented with 1% (w/v) galactose, lactose, melibiose, and raffinose. This was in agreement with the results previously reported for the production of *α*-Gal by* A. fumigatus* [42, 44],* Trichoderma reesei* [43],* Penicillium simplicissimum* [45], and* Humicola* sp. [46]. In previous research work, agricultural waste materials like soya bean flour or soya meal (SM) extract, wheat flour [41], and so forth and complex carbohydrates like GG [18] were used as carbon source in the medium for *α*-Gal production. GG as carbon source was reported to enhance enzyme production in* Bacillus megaterium* VHM1 [47]. The growth pattern of* T. evansii* in culture broth containing different carbon sources after 11 days of incubation at 28°C has been displayed (Figure 1).

**Figure 3:**
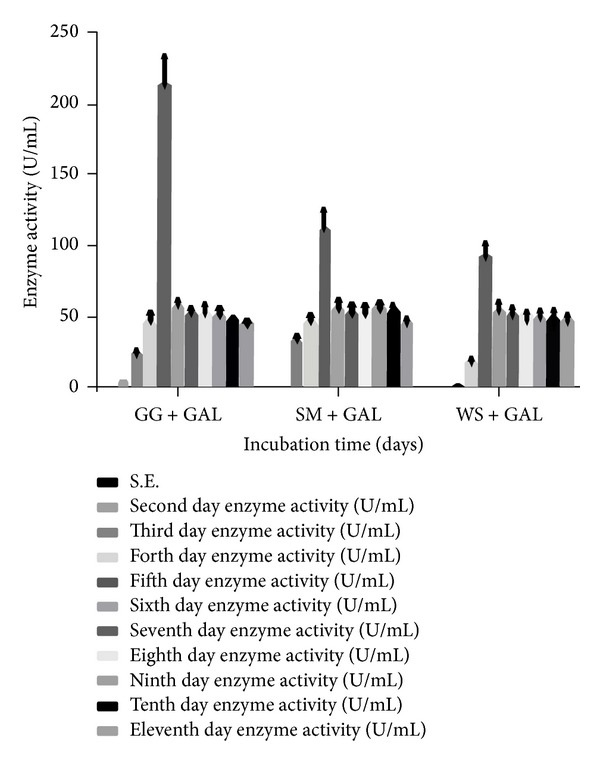
Effect of incubation time and different combinations of carbon sources on the production of *α*-Gal activity (U/mL) secreted by* T. evansii* (WF-3). GG + GAL = guar gum + galactose, SM + GAL = soya bean meal, and WS + GAL = wheat straw + galactose. The experiments have been conducted three times and the values are shown as mean ± SD. Blank bar = not detected (for first day enzyme activity).

When different carbon sources were tested in combination, the activity of *α*-Gal was found to be maximum with GG + GAL (213.63 U/mL) followed by SM + GAL (113.18 U/mL) and WS + GAL (93.07 U/mL). In presence of GG + GAL and SM + GAL, the enzyme secretion was the maximum on the 5th day whereas WS + GAL registered maximum enzyme secretion on the 4th day (Figure 3). Earlier reports have indicated that galactose may prove to be a good inducer for the highest enzyme production (96.70 U/mL) after 2 days of incubation period, followed by melibiose and raffinose [42, 44]. This was in agreement with the previously reported data for the production of *α*-Gal by* A. fumigatus* [42, 44],* Trichoderma reesei* [43], and* Penicillium simplicissimum* [45]. Therefore, microbial cells can be stimulated to produce inducible enzymes in presence of simple sugars which act as inducers. Inducing effect of galactose in the biosynthesis of *α*-Gal in SSF was previously reported by Shankar et al. [48].

**Figure 4:**
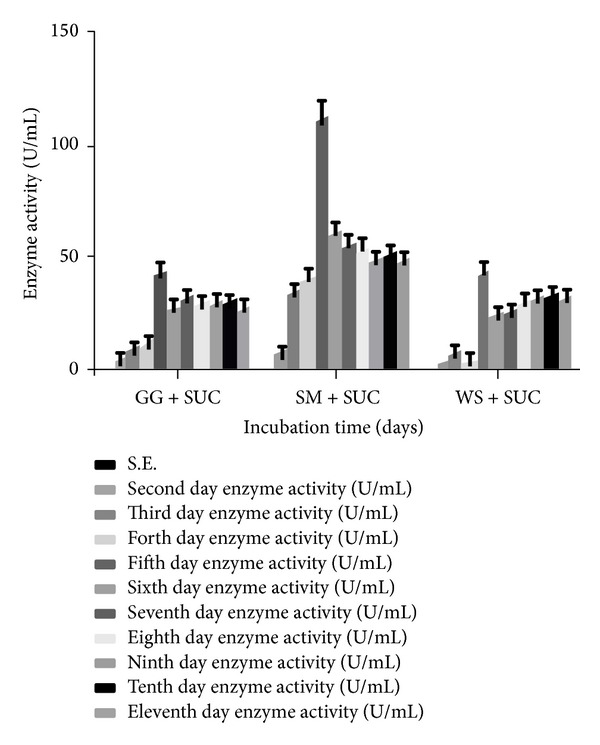
Effect of incubation time on the production of *α*-Gal by* T. evansii* in presence of different combinations of carbon sources. GG + SUC = guar gum + sucrose, SM + SUC = soya bean meal + sucrose, WS + SUC = wheat straw + sucrose, and SUC = sucrose. The experiments have been conducted three times and the values are shown as mean ± SD. Blank bar = not detected (for first day enzyme activity).

While knowing the fact that the monosaccharide requirement for *α*-Gal induction was not only specific for D-galactose, it can be applied for other sugars as well. In another similar experiment, when SM, GG, and WS were combined with sucrose (Suc) in basal culture media,* T. evansii* displayed maximum secretion of enzyme on the 5th day, the extent of secretion being the highest in presence of SM + Suc followed by GG + Suc and WS + Suc (Figure 4). Usually, only the substrates of an enzyme or its structural analogs are able to act as inducers. Dey and Pridham [49] showed that the rate of hydrolysis of a substrate or its binding with *α*-Gal depends upon the substrate having a pyranose ring structure and that the configuration of carbon atoms 1–4 must be similar to that in D-galactose [1]. Addition of sucrose in present study, however, showed significantly lower enzyme activity, the values being 42.26 U/mL, 110.88 U/mL, and 42.66 U/mL when added with GG, SM, and WS, respectively, as compared to the carbon sources alone in media (Figure 2). Surprisingly, in this investigation, sucrose and galactose, when added alone in culture media as carbon sources, sustained substantial growth, but galactose was almost as poor inducer as lactose [45]. This could be due to the presence of invertases, which hydrolyse the sucrose and produce simple sugars in combination with background *α*-Gal. These sugars could then be used for the production of mycelia mass but were unable for any further inducing *α*-Gal production.

### 3.3. Effect of pH and Incubation Temperature on the Enzyme Production by* T. evansii*

**Figure 5:**
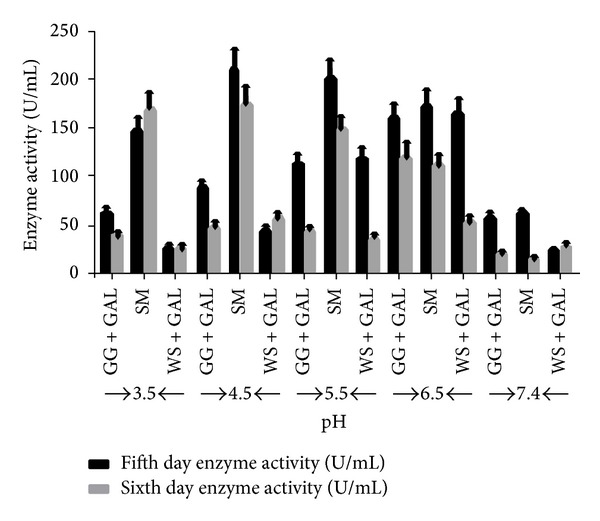
Effect of pH on the production of *α*-Gal by* T. evansii* in presence of different combinations of carbon sources. Culture incubation time was 5 and 6 days with maximum enzyme activity as compared to other incubation days. GG + GAL = guar gum + galactose, SM = soya bean meal, WS + GAL = wheat straw + galactose, and GAL = galactose. The experiments have been conducted three times and the values are shown as mean ± SD.

Generally, fungal and yeast *α*-Gal have optimum pH value in the acidic range varying from 3 to 5 [18, 43, 47], but for some fungal strains extracellular *α*-Gal has been known to work effectively at broad pH range as reported for different fungal genera like* Aspergillus fumigatus*,* P. purpurogenum*, and* T. lanuginosus* [44, 45, 50]. In present study, the enzyme secreted by* Trichoderma evansii* (WF-3) in the culture media containing SM displayed maximum enzyme activity in the pH range of 3.5 to 5.5 on days 5 and 6 in different culture broths (Figure 5). Interestingly, at the physiological pH 7.4, there is considerable reduction in enzyme activity.

**Figure 6:**
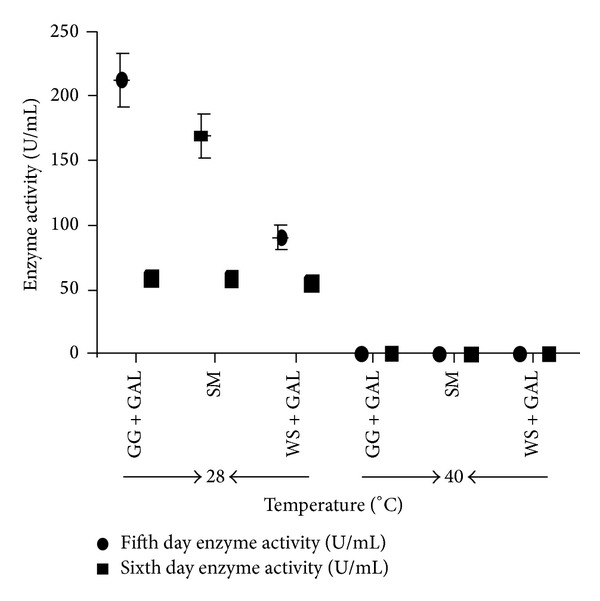
Effect of temperature on production of *α*-Gal activity on the fifth and sixth days of incubation in basic culture medium containing different combinations of carbon sources. Two different temperatures, that is, 28°C and 40°C, have been employed. GG + SUC = guar gum + sucrose, SM + SUC = soya bean meal + sucrose, WS + SUC = wheat straw + sucrose, and SM = soya meal. The culture conditions were the same as described in Section 2. The experiments have been conducted three times and the values are shown as mean ± SD.

The temperature stability of the enzyme is based on the culture habit of the organisms. Obviously, exhibited temperature stability is because the fungi were mesophilic in nature. Effect of incubation temperature on *α*-Gal activity was investigated by incubating standard basal media with inoculums at two different temperatures (28°C and 40°C). The results obtained from the present study showed the preference of 28°C over 40°C for optimum enzyme production (Figure 6). Shivam in his report has suggested that the longer fermentation time was required to attain maximum enzyme production when incubation was carried out at 20°C and 25°C. Because of the mesophilic nature of fungi, the gradual rise in temperature above 35°C concomitantly decreases *α*-Gal production [51]. The results from the present study reflected almost nil enzyme activity when culture media weer placed at 40°C of incubation temperature (Figure 6). The protein secretion, however, under this condition was found to be increased on day 6 with each substrate, the value being maximum with SM (Figure 6).

### 3.4. Effect of Amount of Substrate Concentration in Culture Media on Enzyme Activity

**Figure 7:**
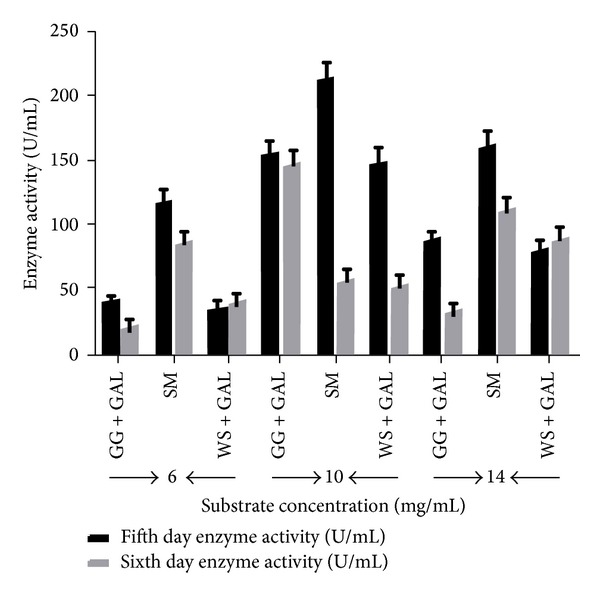
Effect of varying concentrations of different substrate combinations on the excretion of *α*-Gal activity by* T. evansii* (WF-3) in the culture medium incubated for fifth and sixth days. GG + SUC = guar gum + sucrose, SM + SUC = soya bean meal + sucrose, WS + SUC = wheat straw + sucrose, and SM = soya meal. The culture conditions were the same as described in Section 2. The experiments have been conducted three times and the values are shown as mean ± SD.

In present study, three different substrate concentrations, that is, 6, 10, and 14 mg/mL, were used to evaluate the optimum substrate utilization in different standardized culture filtrates, that is, GG + GAL, SM, and WS + GAL, for maximum enzyme production in media. The results from present study indicated that 10 mg/mL of substrate concentration was maximally utilised for optimum enzyme production in media on the fifth day of culture incubation at 28°C, where SM has shown maximum enzyme recovery, that is, 213.02 U/mL, followed by (GG + GAL) (154.02 U/mL) and then with (WS) (148.86 U/mL), while (14 mg/mL) shows decreasing trend, that is, (161.77 U/mL) in (SM), (87.14 U/mL) followed by (79.36 U/mL) in (WS) media and the diluted culture media that is, (6 mg/mL) substrate concentration showed much reduced activity profile as presented in (Figure 7). Some workers demonstrated that considerable increase in reaction efficiency can be achieved by operating at higher GG concentrations than those used in most previous studies (<1%) [52, 53]. Furthermore, those reaction systems such as semisolid particulates can be utilized, with consequent improvements in process ability and decreases in reaction volume compared with dilute viscous solutions. One inference which can be drawn from the present study, however, is that the reduction in reaction efficiency at very high (>40% w/w) GG concentration is not due to irreversible enzyme inactivation [54].

### 3.5. Effect of Different Metal Ions and Inhibitors on Relative Activity of *α*-Galactosidase Activity

Since the metal ions are reported to influence the activity of carbohydrate degrading enzymes, an endeavour was made to evaluate the effect of mono- and divalent metal ions on the activity of *α*-galactosidase. The results presented in Table 1 demonstrated that the monovalent metal ion Ag^+^ could reduce the activity of the enzyme by 60% when used up to 0.1 mM. At the concentration >0.1 mM, it completely inhibited the enzyme activity. In a report by Rudra et al. [55], these metal ions, excluding Hg, have been shown to exert only mild inhibitory effect [53]. However, Park et al. have demonstrated that, in* P. purpurogenum*, the enzyme was strongly inhibited by Hg^2+^ and Ag^+^ ions [56]. In the absence of a thiol group at the active site, the inhibition by Ag^+^ may possibly be attributed to its combination with carboxyl or histidine residue or both. This possibility has been suggested by Dixon and Webb [57] and Myrback and Kemi [58]. Among the divalent metal ions tested, Hg most strongly inhibited the enzyme activity as, at 0.05 mM, no activity was detected. Inhibition by Hg^2+^ usually suggests reaction with thiol groups. However, Webb discussed the coordination of Hg^2+^ between carboxyl and amino groups [59]. Hg^2+^ is also known to react with amino and imidazolium groups of histidine [60] and with peptide linkages [59]. However, Cu^2+^ at 1 and 2 mM concentrations caused 27 and 61% enzyme inhibition, respectively. At 5 mM concentration, Cu^2+^ completely inhibited the enzyme activity. *α*-Gal is highly sensitive to heavy-metal ions such as Ag^+^ and Hg^2+^ but much less sensitive to Cu^2+^. In contrast, Mg^2+^ caused only mild reduction in the enzyme activity (0–20%) at 1, 2, and 5 mM concentrations (Table 1).

The effects of couple of chelators such as EDTA and succinic acid were also monitored on the activity of *α*-Gal. The results presented in Table 3 indicated that EDTA at 0.05 to 0.5 mM concentrations caused only about 50% activity. On the other hand, succinic acid even at very high concentration (10 mM) could reduce the enzyme activity up to 13% only (Table 1). Recently, Rudra et al. [55] have indicated that EDTA was not exerting any inhibitory effect on the enzyme activity from* P. purpurogenum* even at 5 mM concentration. The exact mechanism of almost no effect of chelators on the activity of *α*-Gal, however, is not known.

### 3.6. Effect of Different Culture Medium on the Secretion of Total Proteins by* T. evansii*

The total protein secreted by* T. evansii* was minored by assaying the protein contents in the culture filtrate up to 11 days. The results shown in Table 2 demonstrated that SM present in basal culture media displayed maximum secretion of proteins followed by GG + GAL, SM + SUC, SM + GAL, GG, GG + SUC, WS, WS + SUC, GAL, SUC, and WS + GAL, the values being 435.92 ± 30.04, 266.33 ± 18.09, 207.28 ± 17.09, 182.16 ± 16.02, 164.57 ± 14.08, 95.47 ± 8.07, 67.83 ± 5.6, 43.96 ± 4.3, 23.86 ± 2.2, 23.14 ± 2.1, and 10.05 ± 9.7 *μ*g/mL, respectively.

The effect of pH, incubation temperature, and substrate concentrations on the level of total protein (*μ*g/mL) secreted by* T. evansii* (WF-3) in the culture filtrate has been shown in Table 3. The data indicated that, at different pH, total protein secreted by* T. evansii* was recorded to be higher on the fifth day of incubation as compared to that of the sixth day except at pH 3.5 and pH 4.5 with GG + GAL combination (Table 3).

When different substrate concentrations were used in the culture filtrate, the total protein in media was recorded to be the maximum on the fifth day compared to the sixth day, the value being the maximum with GG + GAL combination. The highest substrate concentration (14 mg/mL) caused reduction in protein synthesis as compared to lower substrate concentrations (6 and 10 mg/mL). The incubation temperature of 28°C was found to be the most suitable for optimum protein synthesis by* T. evansii*; the values of total protein were recorded to be higher on the sixth day compared to higher temperature, that is, 40°C (Table 3).

## 4. Conclusion

The *α*-Gal (*α*-D-galactoside galactohydrolase, EC 3.2.1.22) from* T. evansii* (WF-3) has been isolated and the optimum culture conditions for the growth of this fungal species have been standardized. The results of the present study indicated that* T. evansii* preferred SM as the best carbon source for optimum enzyme production in culture media. The optimum incubation time was on days 5 and 6.* T. evansii* exhibited preference to produce maximum enzyme in culture filtrate at pH 4.5–5.5 when incubated at 28°C. However, it lost activity completely at 40°C. Galactose and sucrose in growth media acted as inducers of enzyme production in GG and WS. SM alone was able to support* T. evansii* to produce maximum enzyme activity. All the carbon sources tested exhibited maximum enzyme production at 10 mg/mL concentration. The optimum secretion of the enzyme from* T. evansii* was dependent on the nature and concentration of carbon source, pH, temperature, and incubation time. This fungal species is expected to be utilized for different industrial and medical purposes. Among the metal ions tested, Hg was found to be the strongest inhibitor of the enzyme followed by Ag and Cu. At high concentration, Mg displayed only mild inhibitory effect. EDTA at very high concentration (10 mM) could inhibit the enzyme activity by 45% only. Succinic acid at this concentration could exert only mild (13%) inhibitory effect.

## Figures and Tables

**Table 1:** Effect of different metal ions on the activity of *α*-Gal secreted by *T. evansii* (WF-3) in culture medium in presence of soya meal (SM) as carbon sources.

Culture media	Metal ions/reagents	Concentration (mM)	Relative Activity (%)
Soya meal (SM)	Cu^2+^	1.0	72.97
		2.0	39.36
		5.0	00.01
	Mg^2+^	1.0	100.0
		2.0	90.31
		5.0	80.28
	Ag^+^	0.05	43.32
		0.10	44.70
		0.20	00.01
		0.50	0
	Hg^2+^	0.05	0
	EDTA	0.05	50.92
		0.10	52.14
		0.20	48.49
		0.50	50.22
	Succinic acid	1.0	80.79
		2.0	79.77
		5.0	80.79
		10	87.18

**Table 2:** Total protein (*μ*g/mL) secreted by *T. evansii* (WF-3) in the culture medium up to 11 days.

Culture media	Total protein (*μ*g/mL) secreted by *T. evansii* (WF-3) in the culture medium up to 11 days										
	Incubation time (days)										
	1	2	3	4	5	6	7	8	9	10	11
GG	164.57 ± 14.08	193.47 ± 15.09	136.60 ± 9.7	103.01 ± 9.8	182.16 ± 17.03	278.38 ± 26.08	265.07 ± 24.06	261.30 ± 25.09	160.80 ± 15.68	28.89 ± 2.69	18.56 ± 1.7
GG + GAL	266.33 ± 18.09	272.61 ± 20.08	321.60 ± 2.2	414.57 ± 33.06	582.91 ± 44.06	312.81 ± 29.85	296.48 ± 28.86	192.16 ± 18.49	159.80 ± 15.34	155.78 ± 14.68	26.89 ± 2.4
GG + SUC	95.47 ± 8.07	90.45 ± 7.6	41.45 ± 3.8	67.83 ± 5.9	35.17 ± 3.2	116.83 ± 10.5	329.15 ± 30.36	590.45 ± 58.78	419.59 ± 40.93	379.39 ± 36.90	331.71 ± 31.08
SM	435.92 ± 30.04	238.69 ± 19.06	314.07 ± 29.08	353.01 ± 30.08	798.24 ± 67.08	361.80 ± 34.05	328.15 ± 29.89	311.56 ± 30.65	291.45 ± 28.78	266.33 ± 25.08	150.01 ± 14.30
SM + GAL	182.16 ± 16.02	90.35 ± 7.8	229.89 ± 20.07	110.55 ± 10.9	239.94 ± 22.05	351.75 ± 34.02	79.15 ± 6.98	107.18 ± 9.89	174.62 ± 16.96	153.26 ± 14.90	160.80 ± 15.56
SM + SUC	207.28 ± 17.09	227.38 ± 20.04	189.69 ± 17.06	189.69 ± 17.68	340.45 ± 32.89	258.79 ± 23.83	242.46 ± 21.98	557.78 ± 53.09	518.84 ± 50.86	383.17 ± 37.68	535.17 ± 51.29
WS	67.83 ± 5.6	114.32 ± 10.89	145.72 ± 13.83	237.43 ± 22.87	329.15 ± 31.31	483.67 ± 46.76	463.56 ± 44.98	356.78 ± 32.88	341.71 ± 33.21	236.18 ± 22.26	230.46 ± 21.86
WS + GAL	10.05 ± 9.7	146.98 ± 13.89	145.30 ± 13.36	301.50 ± 29.76	146.98 ± 13.68	282.66 ± 27.60	214.82 ± 20.96	288.94 ± 27.27	417.08 ± 40.14	396.98 ± 38.36	160.80 ± 15.70
WS + SUC	43.96 ± 4.3	48.99 ± 4.3	74.12 ± 7.12	228.64 ± 21.75	271.35 ± 26.67	237.43 ± 22.80	194.72 ± 16.86	127.89 ± 11.87	635.67 ± 61.64	611.80 ± 60.68	569.78 ± 54.06
GAL	23.86 ± 2.2	67.83 ± 5.3	336.68 ± 32.39	395.72 ± 37.64	201.23 ± 19.63	37.40 ± 35.45	N/D	N/D	N/D	12.56 ± 1.1	N/D
SUC	23.14 ± 2.1	50.25 ± 4.8	60.30 ± 59.86	60.30 ± 5.9	37.68 ± 36.08	50.30 ± 4.9	115.57 ± 10.67	173.36 ± 15.46	152.01 ± 13.83	100.50 ± 9.78	231.46 ± 22.34

GG: guar gum, GG + GAL: guar gum + galactose, GG + SUC: guar gum + sucrose, SM: soya bean meal, SM + GAL: soya bean meal + galactose, SM + SUC: soya bean meal + sucrose, WS: wheat straw, WS + GAL: wheat straw + galactose, and WS + SUC: wheat straw + sucrose. The culture conditions were the same as described in Section 2. The experiments have been conducted three times and the values are shown as mean ± SD.

**Table 3:** Effect of pH, incubation temperature, and substrate concentration on the level of total protein (*μ*g/mL) secreted by *T. evansii* (WF-3) in the culture filtrate.

Culture parameters		Culture media with different carbon sources	Total protein (*μ*g/mL) at 5th day of incubation	Total protein (*μ*g/mL) at 6th day of incubation
pH	3.5	GG + GAL	160.80 ± 15.07	173.36 ± 16.28
		SM	87.93 ± 7.9	70.35 ± 6.8
		WS + GAL	103.02 ± 9.8	86.68 ± 8.6
	4.5	GG + GAL	105.52 ± 9.9	173.36 ± 15.89
		SM	250.00 ± 24.9	133.17 ± 12.75
		WS + GAL	246.23 ± 22.8	244.97 ± 23.9
	5.5	GG + GAL	204.77 ± 19.7	139.45 ± 12.98
		SM	62.81 ± 4.7	116.83 ± 15.43
		WS + GAL	245.16 ± 24.09	247.53 ± 23.69
	6.5	GG + GAL	223.61 ± 21.05	134.42 ± 11.78
		SM	265.07 ± 25.08	164.57 ± 15.07
		WS + GAL	360.55 ± 34.35	59.05 ± 55.98
	7.4	GG + GAL	283.91 ± 25.86	38.53 ± 3.2
		SM	114.32 ± 10.76	61.55 ± 5.9
		WS + GAL	370.60 ± 35.68	209.79 ± 20.03

Substrate conc. (mg/mL)	6	GG + GAL	432.16 ± 42.14	203.52 ± 19.56
		SM	104.27 ± 9.45	76.63 ± 6.8
		WS + GAL	305.27 ± 29.67	82.91 ± 8.0
	10	GG + GAL	476.13 ± 46.43	180.90 ± 16.68
		SM	361.80 ± 34.09	457.28 ± 43.04
		WS + GAL	146.98 ± 12.86	237.44 ± 22.14
	14	GG + GAL	305.28 ± 28.05	80.40 ± 7.8
		SM	193.46 ± 17.06	95.47 ± 8.8
		WS + GAL	87.93 ± 7.6	38.94 ± 3.3

Incubation temperature (°C)	28	GG + GAL	192.16 ± 18.03	582.91 ± 54.24
		SM	361.80 ± 35.64	798.24 ± 70.43
		WS + GAL	146.98 ± 13.43	282.66 ± 23.87
	40	GG + GAL	99.24 ± 9.3	193.49 ± 18.54
		SM	31.40 ± 2.8	34.53 ± 3.3
		WS + GAL	70.35 ± 6.4	82.35 ± 8.0

GG: guar gum, GG + GAL: guar gum + galactose, GG + SUC: guar gum + sucrose, SM: soya bean meal, SM + GAL: soya bean meal + galactose, SM + SUC: soya bean meal + sucrose, WS: wheat straw, WS + GAL: wheat straw + galactose, and WS + SUC: wheat straw + sucrose. The culture conditions were the same as described in Section 2. The experiments have been conducted three times and the values are shown as mean ± SD.
